# Associations of discrimination and pain among older adults: Results from the Einstein Aging Study

**DOI:** 10.1093/geroni/igaf122.3416

**Published:** 2025-12-31

**Authors:** Linying Ji, Monique Balthazar, Yuqi Shen, Margeaux Gray, Richard Lipton, Jennifer Graham-Engeland, Carol Derby, Orfeu Buxton

**Affiliations:** Montana State University, Bozeman, Montana, United States; The Pennsylvania State University, University Park, Pennsylvania, United States; The Pennsylvania State University, University Park, Pennsylvania, United States; The Pennsylvania State University, University Park, Pennsylvania, United States; Albert Einstein College of Medicine, Bronx, New York, United States; The Pennsylvania State University, University Park, Pennsylvania, United States; Albert Einstein College of Medicine, Bronx, New York, United States; The Pennsylvania State University, University Park, Pennsylvania, United States

## Abstract

Chronic pain among older adults varies by race and sex, suggesting the value of examining social determinants (e.g. discrimination) that may drive pain-related outcomes. We examined how discriminatory experiences influence pain reported in daily life and whether those associations vary by race and sex. Participants (N = 296, M_age=77.5 years, 69% women; 46% nonhispanic White, 41% nonhispanic Black, 13% Hispanic/other) are from the Einstein Aging Study. Using ambulatory mobile assessments (6 daily, 2 weeks), person-level average scores were estimated for pain intensity ( mean level, peak), pain interference with cognition (PIC), pain interference with function (PIF). A modified Williams’ Everyday Discrimination questionnaire assessed the frequency and number of reasons for discrimination. Linear regressions, stratified by race and sex, were used to test associations between pain and discrimination, controlling for age, education, BMI, pain medication use, and chronic diseases. Among women, discrimination frequency was associated with PIF; number of reasons for discrimination was associated with average pain intensity and PIF. For men, discrimination frequency was associated with PIC. Among Blacks, discrimination frequency was associated with peak pain and PIC; number of reasons for discrimination was associated with pain intensity and PIF. For White participants, discrimination frequency (not number of reasons for discrimination) was associated with average pain intensity, PIC, and PIF. Discriminatory experiences are associated with increased pain reports that differ by race and sex. Discriminatory experiences had stronger associations with pain for women than men. Number of reported reasons for discriminatory experiences was more strongly associated with pain outcomes in Black participants.

